# Fracture Mode Transition and Energy Dissipation of Brittle Coal Under Confinement Induced by a Flexible Polyurea Coating

**DOI:** 10.3390/polym18121538

**Published:** 2026-06-20

**Authors:** Shan Ning, Weibing Zhu, Biao Fu, Pengjun Gao, Zishuo Jia

**Affiliations:** 1 School of Mines, China University of Mining and Technology, Xuzhou 221116, China; shan.ning@cumt.edu.cn (S.N.); cumtgpj@cumt.edu.cn (P.G.); ts23020021a31@cumt.edu.cn (Z.J.); 2 State Key Laboratory for Fine Exploration and Intelligent Development of Coal Resources, China University of Mining and Technology, Xuzhou 221116, China; 3 General Technology Group Engineering Design Co., Ltd., Jinan 250031, China; fubiao1203@126.com

**Keywords:** polyurea coating, brittle coal, passive confinement, fracture mode transition, energy dissipation

## Abstract

Brittle geomaterials such as coal and rock are prone to unstable failure under high stress and dynamic disturbances, where rapid release of stored elastic strain energy can trigger dynamic disasters. Polyurea, a high-strength and high-ductility elastomer, can form a continuous flexible coating on the surface of coal/rock to regulate their deformation–fracture behavior. Here, uniaxial compression tests were performed on coal specimens coated with polyurea layers of different thicknesses (0–1.25 mm). Acoustic emission (AE) and digital image correlation (DIC) were jointly employed to characterize macroscopic deformation, microcrack evolution, fracture-mode transition, and energy partitioning. The results show that polyurea provides passive lateral confinement that suppresses lateral expansion and shifts macroscopic failure from brittle splitting to progressive ductile damage. AE-based AF–RA analysis indicates that thicker coatings increase the normal stress and shear resistance along potential fracture planes, promoting a microfracture transition from shear-dominated to tension-dominated cracking. Energy analysis demonstrates that the coating enhances pre-peak energy dissipation via coordinated deformation with the coal, while thicker coatings (≥1.00 mm) exhibit pronounced post-peak elastic tensile deformation to absorb and buffer fracture-released energy, impeding the instantaneous energy release typical of bare coal. Moreover, the elastic energy index shows that polyurea markedly reduces impact tendency, with an appropriate thickness stabilizing specimens from strong to weak/non-impact propensity. These findings clarify the coupled confinement–fracture–energy regulation mechanisms of polyurea coatings and provide quantitative guidance for coating-thickness design to mitigate dynamic failure hazards in brittle materials.

## 1. Introduction

Flexible polymer coatings (e.g., polyurea) provide an effective strategy to tailor the failure behavior of brittle substrates via interfacial bonding and confinement [1,2]. Rather than acting as a passive protective layer, the polymer can actively modulate crack initiation, propagation, and coalescence, thereby altering fracture modes and energy dissipation. Quantitatively linking coating-induced constraint to fracture mode transition and energy partitioning is therefore critical for rational design of functional polymer–brittle material systems.

In deep underground engineering, high-stress rock masses may undergo brittle failure, leading to rock bursts, a typical dynamic disaster characterized by strong suddenness and severe destruction [3,4,5]. Its essence is the continuous accumulation of elastic energy in the coal and rock mass during the loading process, and then its concentrated release occurs at the moment of instability [6,7]. Accordingly, the effective brittleness of the coal mass, the inhibition of elastic energy accumulation and the promotion of energy dissipation during the failure process have become the core issues in the research on prevention and control of deep dynamic disasters [8,9].

Generally, existing support systems primarily prevent deep coal and rock dynamic disasters by imposing rigid constraints on the surrounding rock mass [10]. In engineering practice, drilling pressure relief (PDR) with the combination of bolt–mesh–cable support is commonly adopted [11], and various support technology systems have been derived, including high preloading support [12,13], long and short anchor cable hierarchical control technology, full-length preloading support [14,15,16] and soft rock multi-level coupling support [17,18,19,20]. These measures have improved the integrity and load-bearing capacity of the surrounding rock mass to some extent. Nevertheless, the overall deformation coordination capacities and energy absorption capacities of these conventional support structures are relatively insufficient [21]. Particularly, when the coal and rock masses undergo lateral expansion, crack penetration and large deformation under high stress or dynamic load, it is difficult to effectively accommodate the kinetic and elastic energy instantaneously released by the brittle failure of the rock mass.

In this context, owing to the advantages of high construction efficiency, strong adhesion and the rapid formation of continuous protective layers, thin-layer spray lining technology has attracted considerable attention [22,23,24,25,26]. However, the performance of Thin Spray-on Liners highly depends on the adopted base materials. Currently, conventional protective coatings mainly include cementitious materials, epoxy resins, and polyurethanes. To visually demonstrate the necessity of material optimization, Table 1 summarizes the key parameter comparisons among various typical protective coatings based on the extensive engineering literature. Cementitious liners exhibit excellent fire resistance and economic feasibility, but their innate brittleness and low elongation limit their capacity to accommodate massive structural deformations [23]. Epoxy resins can provide high continuous tensile strength and adhesion, but their low elongation at fracture (typically less than 10%) renders them prone to brittle cracking under dynamic loads or impact ground pressure. Although polyurethanes demonstrate satisfying flexibility and deformation capability, their curing process is highly sensitive to underground humidity, often resulting in foaming, which significantly weakens the ultimate mechanical strength and energy absorption capacity.

As a high-performance elastomeric material, polyurea exhibits both high tensile strength and rapid curing characteristics, and the corresponding elongation at fracture usually exceeds 300% [40,41,42,43]. Notably, when polyurea is sprayed on the surface of coal and rock mass, a continuous and dense flexible coating layer is generated [44,45,46]. This reveals that this coating layer can alter the failure and energy dissipation behavior of coal and rock mass via physical mechanisms, such as surface coating constraint, crack bridging and large deformation energy absorption. Existing studies have demonstrated that polyurea coating can not only significantly improve the residual load-bearing capacities of coal and rock pillars [47] but also exhibit excellent fragment constraints and kinetic energy absorption capacities in drop-weight impact [48], explosive loading and dynamic rock burst testing [49].

The effectiveness of polyurea in improving the impact and fracture resistance of coal and rock materials has been validated, but the understanding of the underlying mechanisms remains insufficient. First, the intervention effects of polyurea coating on the microscopic fracture evolution mechanism of coal and rock masses have not been fully elucidated. Moreover, existing studies are mainly dependent on the analysis of macroscopic stress–strain curves, observations of ultimate failure morphologies, or numerical simulations to explain the reinforcement effects of polyurea, but these approaches cannot clearly reveal how a coating alters the initiation and propagation paths of internal original microcracks. Second, the effect of polyurea coating thickness on energy dissipation is not yet explicitly clarified. In general, the energy absorption capacity of polyurea is broadly described as “the increased total energy dissipation,” while quantitative and process-based analysis remains lacking. Additionally, a lack of understanding of the microscopic fracture mechanism renders the interpretation of the polyurea constraint effect in a “black box” state, while the ambiguity of energy evolution and key influencing parameters hinders in-depth research on the impact resistance mechanisms and the optimization design of engineering parameters. In fact, crack propagation is essentially driven by energy, while the fracture mechanism is inseparably coupled with the energy conversion process. Therefore, it is crucial to conduct a collaborative study through a combination of the microscopic fracture mechanism and macroscopic energy evolution.

Thus, the purpose of this work was to investigate the effect of polyurea coatings of various thicknesses on the mechanisms of fracture and energy absorption in brittle coal under compressive loading, as well as to develop criteria for optimizing coating parameters to reduce the risk of dynamic failure. With the use of the DIC technique, the deformation and rebound characteristics of coal samples were quantitatively analyzed. In addition, with the combination of AE signal evolution and the AF-RA parameter criterion, the initiation and propagation of internal microcracks and the transition process of the tension–shear fracture mechanism in the coal sample were comprehensively analyzed. On this basis, the regulatory effects of polyurea coating thickness on the deformation–failure behavior and energy partitioning of coal samples were symmetrically explored using energy analysis methods. Meanwhile, the elastic energy index WET was adopted to evaluate the effectiveness of coatings with different thicknesses in the improvement of impact tendency.

## 2. Materials and Methods

### 2.1. Fundamental Mechanical Properties of Polyurea Coating Material

The polyurea used for the coating preparation in this experiment was provided by Shenzhen Gaodun New Materials Co., Ltd., Shenzhen, China. Table 1 lists the fundamental mechanical properties of polyurea coating material. As shown in Table 2, this material exhibits high tensile strength and excellent tensile deformation capacity, while the corresponding tensile strength reaches 25.5 MPa, which far exceeds the uniaxial compressive strength of typical coal samples. Additionally, the elongation at fracture reaches 247.0%, and a formation of continuous and dense flexible coating can be observed on the surface of fractured coal samples.

### 2.2. Experimental Scheme and Sample Preparation

To explore the effects of polyurea coating thickness on the mechanical response and failure characteristics of coal samples, five coating thickness levels were selected, namely 0.25, 0.50, 0.75, 1.00 and 1.25 mm, while uncoated bare coal samples were also prepared as a control group. Specifically, the uncoated bare coal samples were designated as C1-C3, and the coated groups were divided into five series (Pu1–Pu5) on the basis of coating thickness. Table 3 lists the grouping and designations of coal samples.

In accordance with the recommendations of the International Society for Rock Mechanics (ISRM), the raw coal was processed into cuboid samples with dimensions of 50 mm × 50 mm × 100 mm. Subsequently, both end faces of the samples were ground using a grinding machine to ensure that the parallelism error cannot exceed ±0.05 mm, while the resulting end faces were perpendicular to the longitudinal axis. Eventually, a total of 18 standard coal samples were fabricated.

To eliminate the influences of moisture and surface pulverization on the coating adhesion, the coal samples were cleaned and dried with anhydrous ethanol before being coated. The coating procedures are described as follows. First, a silane coupling agent was used on the sidewalls and end edges of coal samples, and then, after 24 h of curing, polyurea was applied to multiple layers on the basis of the target thickness. After each coating was completed, an interval of 4 h was applied for surface drying. To accurately determine the thickness and ensure the uniformity of the polyurea coating, a rigorous multi-point measurement average method was adopted. Prior to coating and after complete curing, the dimensions of each cuboid specimen were precisely measured using a digital vernier caliper. The measurements were conducted at three specific cross-sections: the two ends (top and bottom) and the middle of the specimen. At each cross-section, two mutually perpendicular side lengths were measured. The actual coating thickness at each point was calculated as half of the difference between the post-curing and pre-coating dimensions. Finally, the ultimate coating thickness for a given specimen was determined by averaging these calculated values from the six measurement dimensions across the three cross-sections. Furthermore, the variance among these measurement points was strictly checked and controlled to evaluate and guarantee the uniform distribution of the polyurea coating along the specimen surfaces. The coated samples were then placed at room temperature and further cured for 24 h to ensure complete solidification.

### 2.3. Test System and Monitoring Equipment

#### 2.3.1. Test System and Scheme

**Figure 1:**
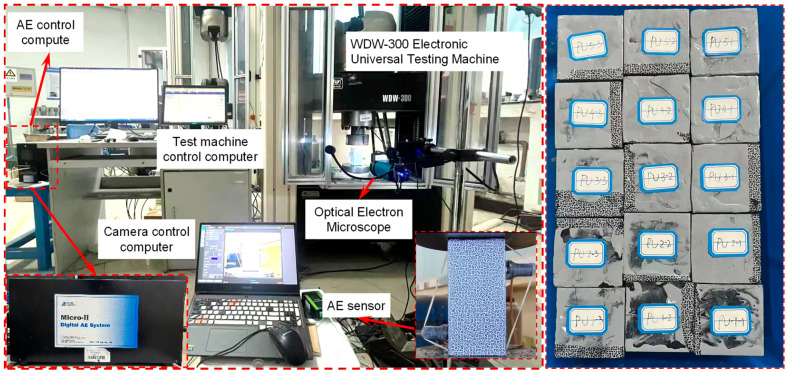
Test system and as-prepared polyurea-constrained coal samples.

All mechanical tests were carried out using a WDW-300 electronic universal testing machine(Changchun Sinter, Changchun, China), and the displacement control mode was used in the uniaxial compression tests with a loading rate of 0.3 mm/min. The test system was mainly composed of a loading device, an AE monitoring system and a DIC image acquisition system. Figure 1 illustrates the test system and the as-prepared polyurea-constrained coal samples.

#### 2.3.2. Acoustic Emission

During the test process, a PCI-2 AE monitoring instrument manufactured by Physical Acoustics Corporation (Princeton Junction, NJ, USA), was used to collect the acoustic wave signals of the coal sample under loading. The AE probe was placed on the unloaded side surface of the coal sample. To improve the acoustic coupling effect between the probe and the coal sample and reduce the signal attenuation, Vaseline was uniformly coated on the contact surfaces as a coupling agent. The preamplifier gain of the system was set to 40 dB, while the sampling frequency was set to 1 MHz.

In this study, to quantitatively characterize the damage evolution of coal samples during the loading process, the AE cumulative count was introduced as the damage evaluation index, and the damage variable (*D*) can be defined by Equation (1), as follows.

(1) $$D=\frac{C_{t}}{C_{0}},$$

where $C_{t}\;$ is the AE cumulative count at time t; $C_{0}$ is the AE cumulative count at the time of the complete failure of the sample.

To ensure the comparability of the damage variable *D* across all specimens, a single quantitative criterion was adopted to define the moment of complete specimen failure. Specifically, for every specimen, regardless of coating thickness, the complete failure moment was determined as the first time the axial stress dropped below 70% of the peak stress.

The b-value of AE can reflect the fracture scale and the corresponding spatial distribution characteristics. Generally, an increase in the b-value of AE demonstrates a more diffuse distribution of microfractures and a higher proportion of small-scale fractures. At the lab scale, the magnitude in the Gutenberg–Richter (G-R) relationship is usually characterized by AE amplitude, while it can be expressed by Equation (2), as follows.

(2) $$\begin{array}{c} lgN=a-b\times \frac{A_{dB}}{20}\\ A_{dB}=20\times lgA_{mV}-A_{pre} \end{array}\},$$

where *N* is the AE cumulative count with an amplitude exceeding *A_dB_*, *A_dB_* is the AE amplitude expressed in decibels (dB), *A_mV_* is the AE amplitude expressed in mV, and *A_Pre_* is the preamplifier gain, which was taken as 40 dB in the experimental groups. During the calculation process, the *A_dB_* interval was set to 1 dB, while the AE events within different amplitude ranges were statistically analyzed, and thus, the b-value can be determined using the least-squares linear regression method.

**Figure 2:**
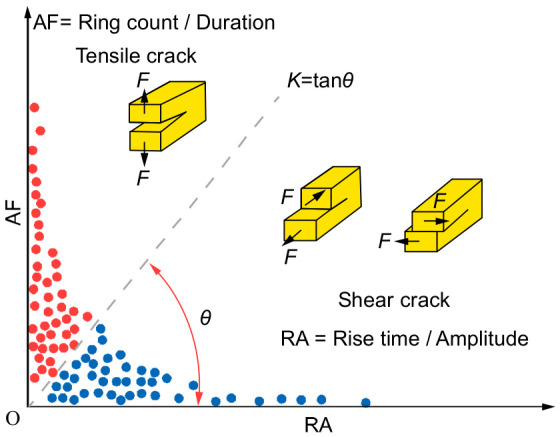
Schematic diagram of AF-RA scatter distribution and crack type identification.

Additionally, to distinguish the AE characteristics under different fracturing mechanisms, the RA value (i.e., rise time/amplitude) and AF value (i.e., average frequency) were adopted to identify crack types. Generally, the tensile cracks often exhibit low RA and high AF, whereas the shear cracks often show high RA and low AF. Based on relevant research results [50], a segmentation slope of K = 11 was selected in this study as the discrimination threshold between tensile cracks and shear cracks. Figure 2 presents the corresponding discrimination principle.

It should be noted that the polyurea coating on the specimen side surface may somewhat attenuate AE signals, particularly for thicker coatings. However, the AF-RA classification method is relatively robust against amplitude attenuation because the RA parameter (rise time divided by amplitude) is a normalized ratio in which amplitude changes affect both the numerator and denominator proportionally. The AF-RA analysis in this study focuses on the relative proportion of tensile and shear cracks across different coating groups rather than absolute signal amplitudes, which further mitigates the impact of systematic signal attenuation. The clear clustering trends observed in the AF-RA scatter plots across all thickness groups confirm that the coating-induced attenuation does not fundamentally alter the fracture mode identification.

#### 2.3.3. DIC Technique

In this study, to achieve the full-field deformation information of the coal sample surface, the DIC technique was adopted for the displacement field analysis. The DIC calculation program was performed using the open-source software Ncorr v1.2.2, based on MATLAB 2023b [51]. By analyzing the images collected during the loading process, the full-field displacement of the coal sample surface can be obtained, including the horizontal displacement U and the vertical displacement V.

To satisfy the demands of the DIC algorithm for image texture features, a random speckle field was prepared on the coal sample surface before the mechanical test. Specifically, the DIC procedures were described as follows. First, a uniform layer of matte white paint was sprayed on the coal sample surface as a base color, and then black paint was randomly sprayed to form the high-contrast speckle field, which can provide the necessary grayscale gradient information for sub-region matching and displacement tracking in the DIC algorithm.

Meanwhile, the image acquisition system was composed of a computer and a GP-S1000 electron microscope. This GP-S1000 electron microscope was manufactured by Kunshan Gaopin Precision Instruments Co., Ltd., Kunshan, China, and was equipped with a 1–14× zoom lens, a CMOS sensor with a resolution of 1920 × 1080 pixels and a high-intensity LED light source. During the test process, the sampling frequency of the microscope was set to 30 frames/s, and thus, the surface deformation and crack evolution characteristics of the coal sample under loading were continuously and clearly recorded.

### 2.4. Energy Monitoring Scheme Based on the Analysis of Stress and Strain

The process of deformation and instability failure in rock masses under loading is essentially derived from the synergistic effects of energy accumulation, dissipation and release. Generally, the total strain energy (U), elastic strain energy ($U_{e}$), and dissipation strain energy ($U_{d}$) can be characterized by the area relationship of the stress–strain curve.

**Figure 3:**
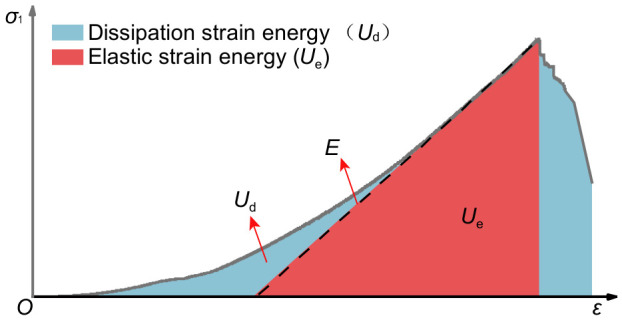
Relationship between elastic strain energy and dissipation strain energy in the rock mass.

Assuming that no heat exchange occurs between the sample and the external environment during the loading process, the corresponding relationships among the various energy parameters are represented in Figure 3.

(3) $$\begin{array}{c} U=U_{e}+U_{d}\\ U_{e}=\frac{1}{2}\sigma_{1}\varepsilon_{1}\approx \frac{\sigma_{1}^{2}}{2E} \end{array}\}$$

where U is the total energy, kJ/m^3^; $U_{e}$ is the elastic strain energy, kJ/m^3^; $U_{d}$ is the dissipated energy, kJ/m^3^; $\sigma_{1}$ is the axial stress measured by the uniaxial compression test, MPa; $\varepsilon_{1}$ is the axial strain at the corresponding time, %; and *E* is the elastic modulus of the sample, GPa.

## 3. Results

### 3.1. Regulatory Effects of Polyurea Coatings on the Macroscopic Mechanical Characteristics of Coal Samples

#### 3.1.1. Stress–Strain–Damage Evolution Characteristics of Coal Samples with Various Polyurea Coating Thicknesses

**Figure 4:**
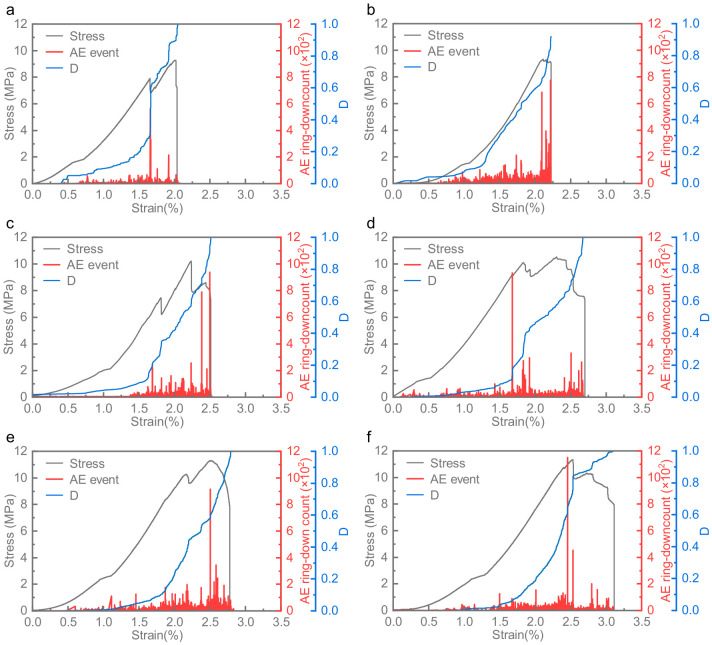
Stress–strain curves and AE damage evolution characteristics of coal samples with various polyurea thicknesses: (**a**) bare coal sample; (**b**) polyurea coating thickness of 0.25 mm; (**c**) polyurea coating thickness of 0.50 mm; (**d**) polyurea coating thickness of 0.75 mm; (**e**) polyurea coating thickness of 1.00 mm; (**f**) polyurea coating thickness of 1.25 mm.

Figure 4 shows the stress–strain–damage curves of coal samples with various polyurea coating thicknesses. It can be seen that the polyurea coatings exhibit a significant regulatory effect on the macroscopic mechanical behavior of coal samples, and thus, the load-bearing capacities and failure characteristics are remarkably enhanced, which can promote a transition in the fracture mode from brittle failure to ductile failure. As presented in Figure 4a, the bare coal sample reaches peak stress at an axial strain of 2.02%, and then the stress is rapidly reduced, indicating obvious brittle failure characteristics. In contrast, when the polyurea coating thickness is increased to 1.25 mm (Figure 4f), the peak strain of the coal sample reaches 2.53%, and the post-peak stress evolution transitions from a sharp decline to a gradual decrease until the occurrence of complete failure at a strain of 3.11%. These results demonstrate that the polyurea coating not only enhances the ultimate load-bearing capacity of the coal sample but also significantly slows down the stress decline rate in the post-peak stage, weakens the brittle instability characteristics, and thus extends the post-peak stage of load-bearing deformation.

With the combination of AE characteristics and damage variable data, it is apparent that when the bare coal sample reaches the peak stress, the AE ringing counts experience a sudden increase, while the damage variable undergoes a synchronous abrupt change and rapidly approaches 1. This phenomenon indicates that the elastic energy accumulated within the bare coal sample is dramatically released, resulting in the instantaneous structural collapse. In contrast, the damage variable of the coal sample with high coating thickness exhibits a gradual accumulation characteristic in the post-peak stage, where a more gradual increase process is observed, and the AE ringing counts remain highly active throughout the entire post-peak stage. This suggests that after the introduction of the polyurea coating, the internal crack evolution of the coal sample transitions from rapid penetration and instability to progressive propagation. Hence, the input energy can be steadily dissipated through sustained fracture, and thus, the instantaneous concentrated release of elastic energy is inhibited in the post-peak stage.

#### 3.1.2. Strength and Deformation Parameter Analysis

**Figure 5:**
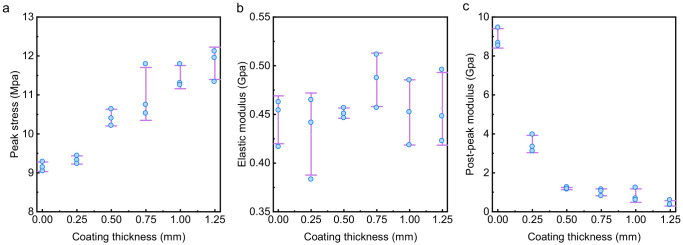
Mechanical parameters of coal samples with different polyurea coating thicknesses: (**a**) peak stress; (**b**) elastic modulus; (**c**) post-peak modulus.

The experimental results demonstrate that the peak load-bearing capacities of the coal samples are significantly enhanced by the introduction of polyurea coatings, and their mechanical behavior can also be affected. As shown in Figure 5a, the peak stress of bare coal samples is only within the range of 9.04–9.28 MPa. After the introduction of polyurea coating, the peak stress of the coal samples exhibits an increasing trend with coating thickness. Specifically, when the polyurea coating thickness reaches 0.50 mm, the peak stress is increased to 10.21–10.64 MPa. When the polyurea coating thickness is within the range of 0.75–1.25 mm, the peak stress of the coal sample is further increased to 10.53–12.13 MPa. This increase in the mechanical strength of the coal sample is primarily derived from the lateral constraint provided by the polyurea coating during the loading process. This constraint effect can significantly suppress the initiation and lateral propagation of microcracks within the coal mass, and thus, the formation of macroscopic fracture surfaces is delayed, leading to the enhanced ultimate load-bearing capacity of the material.

Unlike the variations in peak stress, the polyurea coating exhibits a negligible effect on the elastic modulus of the coal samples in the initial loading stage. As presented in Figure 5b, the statistical data demonstrate that the elastic modulus of each group of coal samples exhibits relatively slight variations within the range of 0.38–0.51 GPa. This phenomenon shows that in the linear elastic stage, no significant lateral expansion or deformation occurs within the coal sample, suggesting that the encapsulation and constraint effects of polyurea coating are not fully activated. Hence, the primary mechanical contribution of polyurea material is not reflected in the improvement of the elastic modulus of the coal sample but rather in the regulation of the deformation and instability process of the coal sample through lateral constraint and synergistic energy dissipation in the peak stress and post-peak stages.

In the post-peak stage, the fracture process of the coal sample is significantly altered by the polyurea coating, and the corresponding fracture mode transitions from brittle failure to ductile progressive failure. To enable a physically meaningful and consistent comparison of the markedly different post-peak behaviors, the post-peak modulus was defined as the absolute value of the secant slope of the descending branch from the peak stress point to the point where the stress decreases to 70% of the peak stress. Figure 5c shows that with the increasing polyurea coating thickness, the post-peak modulus of the coal sample is gradually reduced. The post-peak modulus of the bare coal sample reaches as high as 8.55–9.48 GPa. Conversely, the post-peak modulus of the coal sample with the polyurea coating is rapidly reduced to 0.35–3.97 GPa. Such a reduction in the post-peak modulus reveals the relatively slow decline rate of the post-peak load-bearing capacity. With the increasing coating thickness, the polyurea material exerts pronounced encapsulation and confinement effects during the crack penetration process within the coal mass. Eventually, this flexible constraint mechanism promotes a more progressive, stable and controllable failure evolution in the coal sample.

### 3.2. Constraint Characteristics of Polyurea Coatings on Fracture Morphologies

#### 3.2.1. Fracture Characteristics of Coal Samples with Various Polyurea Coating Thicknesses

**Figure 6:**
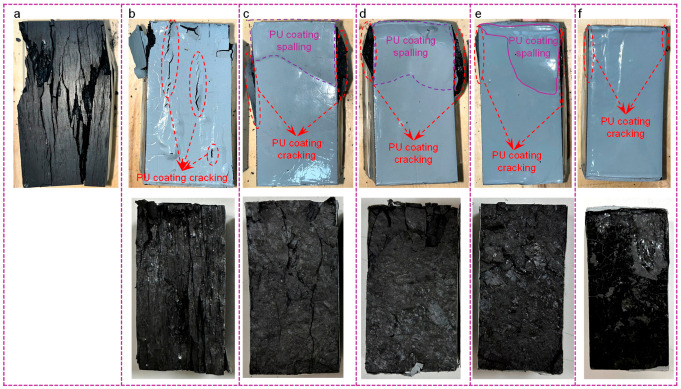
Fracture characteristics of coal samples with various polyurea coating thicknesses (including external surface morphologies and internal cross-section views): (**a**–**f**) fractures of coal samples with polyurea coating thicknesses of 0 mm, 0.25 mm, 0.50 mm, 0.75 mm, 1.00 mm and 1.25 mm, respectively.

To further reveal the regulatory effects of polyurea coating thicknesses on the fracture modes of coal samples, a comparative analysis of the fracture morphologies of coal samples with various polyurea coating thicknesses was conducted in this section. As presented in Figure 6a–f, the fracture morphologies and post-fracture structural integrity of the coal samples are significantly altered by the polyurea coating, and these effects are progressively enhanced by the increased coating thickness. The bare coal sample exhibits fragmentation into multiple blocks after the occurrence of fracturing, indicating a typical brittle splitting fracture mode. When the coating thickness reaches 0.25 mm, multiple cracks and localized cracking can still be observed on the sample surface, suggesting that the polyurea coating can synchronously deform and fracture with the coal mass in the rapid crack propagation stage. Meanwhile, coating thickness exerts a limited constraint effect on both crack propagation and fragment detachment.

When the coating thickness is increased to 0.50–1.00 mm, the coal sample surface remains relatively intact. No obvious crack penetration can be detected in the main body of the coating, while the cracking or spalling only occurs at the edges and ends of the cuboid sample. This phenomenon is closely associated with the geometric configuration of the coal sample, where the coating thickness is relatively thin at the edges, and localized stress concentration appears in the edge regions; thus, these regions are prone to becoming the preferential sites for failure initiation. Nevertheless, within this thickness range, the coal sample surface is maintained with relatively good integrity. This demonstrates that the polyurea coating thickness within this range can provide encapsulation and constraint effects, and thus, the ejection and detachment of coal fragments are inhibited.

To further reveal the authentic damage state beneath the coating, internal cross-section views of the post-test samples were additionally captured and are presented in Figure 6. The internal observations intuitively display a progressive transition from severe fragmentation to relative core integrity as the coating thickness increases. For the bare coal and the thinly coated specimens (0.25 mm), the coal matrix is completely cut by multiple deep principal cracks, confirming thorough structural collapse. When the coating thickness is increased to 0.75–1.25 mm, the internal damage profile undergoes a fundamental transformation. Instead of forming intersecting fragmented networks, the internal structure maintains a high degree of continuity.

#### 3.2.2. Deformation Characteristics of Polyurea Constraint Tests

**Figure 7:**
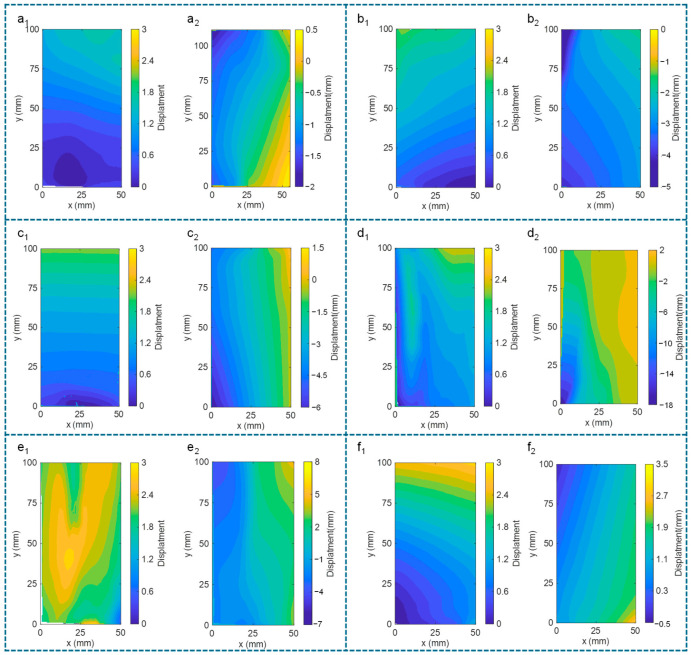
Vertical and horizontal displacement contour maps of coal samples with different polyurea coating thicknesses prior to failure: (**a1**,**a2**) vertical and horizontal displacement contour maps of bare coal sample, respectively; (**b1**,**b2**) vertical and horizontal displacement contour maps of coal sample with coating thickness of 0.25 mm, respectively; (**c1**,**c2**) vertical and horizontal displacement contour maps of coal sample with coating thickness of 0.50 mm, respectively; (**d1**,**d2**) vertical and horizontal displacement contour maps of coal sample with coating thickness of 0.75 mm, respectively; (**e1**,**e2**) vertical and horizontal displacement contour maps of coal sample with coating thickness of 1.0 mm, respectively; (**f1**,**f2**) vertical and horizontal displacement contour maps of coal sample with coating thickness of 1.25 mm, respectively.

The deformation characteristics of coal samples are altered by the lateral constraint effect of polyurea coating. Accordingly, the final displacement contour maps of coal samples with different coating thicknesses prior to failure were analyzed, and Figure 7 presents the corresponding results. By comparing the horizontal and vertical displacement contour maps of coal samples with various polyurea coating thicknesses, it can be seen that the coating thickness has a significant impact on the deformation evolution and instability mode of coal samples.

As illustrated in Figure 7(a1–f1), the vertical displacement contour maps and monitoring data demonstrate that the axial deformation of the coal sample is increased by the increased polyurea coating thickness. The maximum displacement of the bare coal sample is only 2.03 mm, indicating the occurrence of fracture under relatively low axial deformation. In contrast, when the polyurea thickness is increased from 0.25 mm to 1.25 mm, the maximum displacement steadily rises from 2.22 mm to 3.10 mm, corresponding to an increase of ~52.7%. This reveals that the rapid propagation and penetration instability of internal cracks within the coal sample are effectively suppressed by the polyurea coating through continuous passive lateral constraint, which can significantly improve the axial compressive deformation limit of the coal sample and promote the fracture mode transition from brittle failure to ductile failure.

As illustrated in Figure 7(a2–f2), the horizontal displacement contour maps intuitively reflect the lateral deformation characteristics of coal samples. As shown in Figure 7(a2), the horizontal displacements of the bare coal sample along the positive and negative directions of the coordinate system can be determined as 0.42 mm and −1.80 mm respectively, and a total lateral deformation of 2.22 mm is obtained. It should be noted that the magnitude of horizontal deformation gradually increased with the increasing coating thickness. When the coating thickness reaches 0.75 mm, the horizontal displacements of the coal sample along the positive and negative directions of the coordinate system can be determined as 6.00 mm and −17.00 mm respectively, and a total lateral deformation of 23.00 mm can be achieved. This result demonstrates that under this coating thickness condition, the polyurea coating can accommodate significant deformation while maintaining the structural integrity of the coal sample.

As presented in Figure 7(e2–f2), when the polyurea coating thickness is further increased to 1.00–1.25 mm, the horizontal deformation value exhibits a declining trend. Taking the coal sample with a coating thickness of 1.00 mm as an example, the corresponding horizontal displacements along the positive and negative directions of the coordinate system are 7.27 mm and −5.92 mm respectively, and the total lateral deformation reaches 13.19 mm. This result demonstrates that when the coating thickness reaches a relatively large level, the polyurea coating can provide a more pronounced lateral constraint effect, and thus, the lateral expansion and propagation of macroscopic cracks within the coal sample can be effectively inhibited.

#### 3.2.3. Unloading Rebound Characteristics of Coal Sample with Large Polyurea Coating Thickness

**Figure 8:**
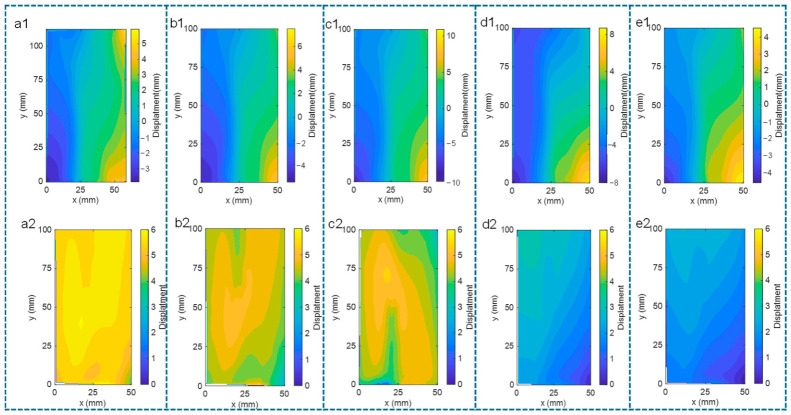
Horizontal and vertical displacement contour maps before and after unloading of coal sample with the polyurea coating thickness of 1.00 mm: (**a1**,**a2**) horizontal and vertical displacement contour maps at 5900 s; (**b1**,**b2**) horizontal and vertical displacement contour maps at 5910 s; (**c1**,**c2**) horizontal and vertical displacement contour maps at 5920 s; (**d1**,**d2**) horizontal and vertical displacement contour maps at 5930 s; (**e1**,**e2**) horizontal and vertical displacement contour maps at 5940 s.

The coal sample with the polyurea coating thickness ≥ 1.00 mm exhibits significant unloading rebound characteristics after failure under the elastic constraint of the polyurea coating. In this study, to elucidate this phenomenon, deformation contour maps of the coal sample with the polyurea coating thickness of 1.00 mm were selected as an example. Figure 8 illustrates the displacement data of the coal sample constrained by polyurea coating within 40 ms before and after final failure and unloading.

Specifically, Figure 8(a1–b2) show displacement contour maps of the coal sample prior to failure, and Figure 8(c1,c2) shows the displacement contour map of the coal sample at the moment of failure. When loaded to the ultimate state, the coal sample exhibits significant deformation, and the vertical displacement reaches 4.42 mm, while the total horizontal displacement is sharply increased to 25.17 mm. As the testing machine begins to unload (Figure 8(d1–e2)), the coal sample exhibits significant rebound, with the vertical displacement rapidly recovering to 2.70 mm and the total horizontal displacement reducing to 18.67 mm. These results demonstrate that the thick polyurea coating still possesses strong elastic recovery capability after failure, which can drive the macroscopic deformation and shrinkage of the coal sample during the unloading process.

The aforementioned phenomenon indicates that the thick polyurea coating still exhibits constraint and buffering effects in the post-peak stage. In the later stage of loading, although the fracture occurs within the coal mass and tends to become structurally unstable, the external thick polyurea coating is not completely fractured, and instead substantial elastic deformation is accumulated during the passive tensile process. When the external load is unloaded, the polyurea coating exhibits a significant shrinkage effect, and the partial closure of previous fracture propagation within the coal mass is facilitated, which drives the overall macroscopic deformation of the coal sample to rebound.

### 3.3. Constraint Effect of Polyurea Coating on Fracture

#### 3.3.1. Fracture Mechanism Analysis Based on AF-RA Distribution

**Figure 9:**
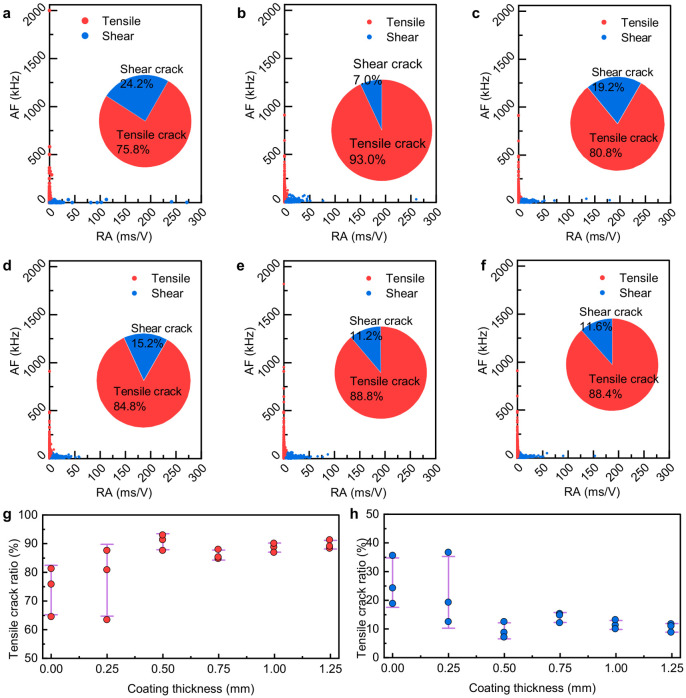
Distributions and statistical results of AF-RA values of coal samples with various polyurea thicknesses: (**a**) bare coal sample; (**b**) polyurea coating thickness of 0.25 mm; (**c**) polyurea coating thickness of 0.50 mm; (**d**) polyurea coating thickness of 0.75 mm; (**e**) polyurea coating thickness of 1.00 mm; (**f**) polyurea coating thickness of 1.25 mm; (**g**) proportions of tensile cracks; (**h**) proportions of shear cracks.

The distribution characteristics of acoustic emission parameters AF and RA were utilized to identify the dominant types of crack propagation within the coal samples. As shown in Figure 9, during the failure process, the AF values are concentrated within the range of 0–500 kHz, while the RA values are concentrated within the range of 0–50 ms/V. For both the bare coal samples and the coal sample with the thin polyurea coating thickness of 0.25 mm, the proportions of tensile cracks and shear cracks exhibit significant fluctuations. As presented in Figure 9g,h, the average value of tensile cracks in the bare coal sample reaches ~73.8%, while the average value of shear cracks is ~26.2%, and the corresponding dispersion is relatively large. In contrast, for the coal sample with the thin polyurea coating thickness of 0.25 mm, the proportion of shear cracks exhibits considerable fluctuations within the range of 12.4%~36.7%.

With the increasing polyurea coating thickness, the proportion of tensile cracks within the coal samples is gradually increased, whereas the proportion of shear cracks is continuously reduced. It is evident that the proportion of tensile cracks in the coal sample with the polyurea coating thickness of 0.50 mm reaches 87.6–93.0%, and an average value can be determined as 90.7%. In addition, the average proportions of tensile cracks in the coal samples with polyurea coating thicknesses of 0.75 mm, 1.00 mm and 1.25 mm remain relatively stable at 86.0%, 88.6% and 89.6%, respectively. Their average proportions of shear cracks are reduced to 9–14%, while the dispersion within the group also declines. These results demonstrate that the relatively thick polyurea coating can provide a more pronounced lateral constraint for the coal samples to increase the normal stress on the potential fracture surface, and thus, the shear slip and propagation of microcracks are suppressed, while the transition of microscopic shear fracture to tensile fracture is promoted in the coal mass.

#### 3.3.2. Relationships Among b-Value, AE Energy and Stress-Time Evolution

**Figure 10:**
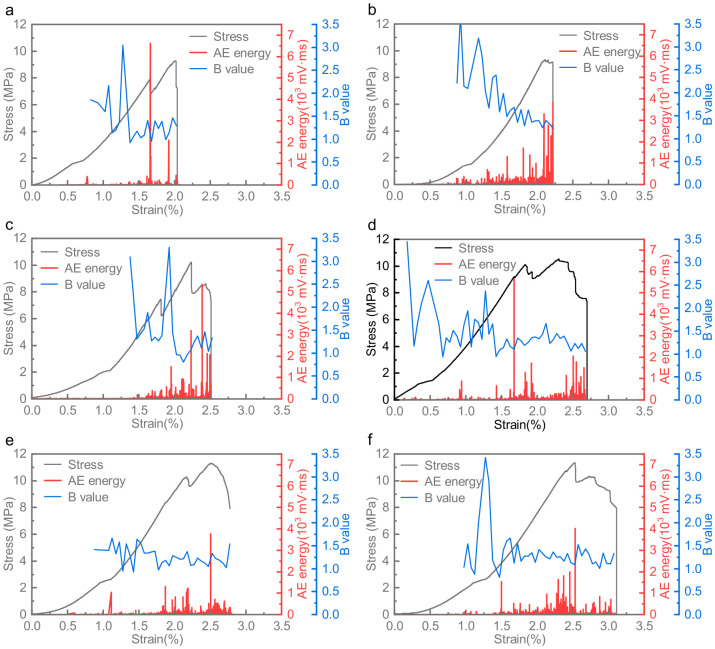
Relationships among stress, AE energy, b-value and strain curve evolution of coal samples with different polyurea thicknesses: (**a**) bare coal sample; (**b**) polyurea coating thickness of 0.25 mm; (**c**) polyurea coating thickness of 0.50 mm; (**d**) polyurea coating thickness of 0.75 mm; (**e**) polyurea coating thickness of 1.00 mm; (**f**) polyurea coating thickness of 1.25 mm.

The progressive failure process within the coal samples from the microcrack initiation and propagation to the macroscopic crack penetration can be characterized by the evolution of the acoustic emission b-value. As illustrated in Figure 10, in the loading stage before reaching peak stress, the b-values of all coal samples exhibit a significant decreasing trend and reach 1.0–1.5 near the peak stress. The rapid decrease in the b-value suggests that the AE activity gradually evolves from numerous low-energy events to a small number of high-energy events, and the internal microcrack propagation is accelerated, leading to the formation of a macroscopic fracture surface.

In the post-peak stage, the b-value of the coal sample does not exhibit a rebound but remains stable within the range of 1.0 and 1.5. When the coating thickness reaches 0.50 mm, a fracture event with a maximum energy of 5.66 × 10^3^ mV·ms still appears in the post-peak stage. However, when the coating thickness is further increased to 0.75 mm, 1.00 mm and 1.25 mm, the maximum energy of AE event in the post-peak stage is reduced to 2.04 × 10^3^ mV·ms, 1.15 × 10^3^ mV·ms and 0.41 × 10^3^ mV·ms, respectively. It is apparent that the intensity of the high-energy post-peak event is attenuated with increasing polyurea coating thickness. Notably, the consistently low b-value in the post-peak stage demonstrates that a relatively complete macroscopic fracture network is formed within the coal sample, while the AE activity mainly corresponds to the frictional sliding along the macroscopic fracture surface. The attenuation of high-energy events indicates that the intense displacement of the fractured coal mass in the post-peak stage is effectively constrained by the thick polyurea coating through the circumferential constraint effect, and thus the intensity of high-energy fracture events is weakened.

### 3.4. Energy Dissipation Characteristics of Coal Samples with Various Polyurea Coating Thicknesses

#### 3.4.1. Evolution Characteristics of Total Strain Energy

**Figure 11:**
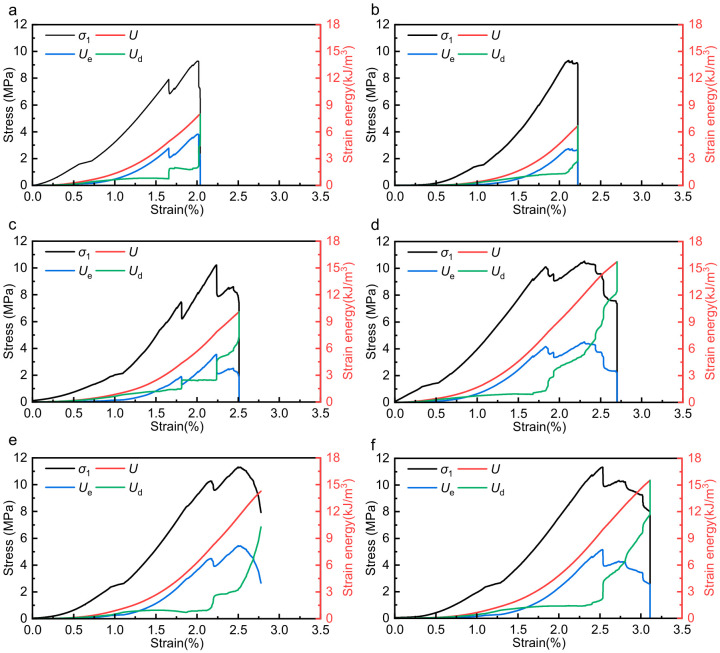
Energy evolution curves of coal samples with various coating thicknesses: (**a**) bare coal sample; (**b**) polyurea coating thickness of 0.25 mm; (**c**) polyurea coating thickness of 0.50 mm; (**d**) polyurea coating thickness of 0.75 mm; (**e**) polyurea coating thickness of 1.00 mm; (**f**) polyurea coating thickness of 1.25 mm.

The introduction of polyurea coating not only significantly enhances the strength and deformation capacity of coal samples by providing effective lateral constraint but also contributes to the energy absorption and dissipation during the compression process. Figure 11 illustrates the energy evolution curves of different coal samples during the compression process. It can be found that the relatively low total strain energy (U) values of the bare coal sample and the coal sample with a thin coating thickness of 0.25 mm can be determined as 7.99 kJ/m^3^ and 6.68 kJ/m^3^, respectively. With increasing coating thickness, the total strain energy (U) of the coal sample is remarkably increased. Particularly, when the polyurea coating thickness reaches 0.50 mm and 0.75 mm, the U value is increased to 10.09 kJ/m^3^ and 15.74 kJ/m^3^, respectively. Moreover, when the coating thickness is increased to 1.0 mm and 1.25 mm, the U value reaches 14.27 kJ/m^3^ and 15.54 kJ/m^3^, respectively, indicating that the enhancement exhibits a gradual trend and generally remains stable. This demonstrates that the polyurea coating can effectively constrain the lateral expansion and deformation of coal and rock mass, and thus, the total external work that the composite sample can absorb before failure is substantially improved.

#### 3.4.2. Energy Partitioning Characteristics in the Pre-Peak Stage

**Figure 12:**
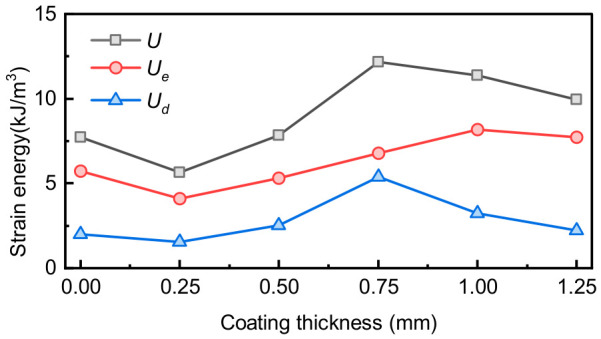
Statistical results of total energy, elastic strain energy and dissipation strain energy at the stress peak point of coal samples with different polyurea coating thicknesses.

Before the coal sample reaches the peak stress, the ratio of elastic energy (Ue) to dissipation strain energy (Ud) varies with the polyurea coating thickness. Figure 12 shows the energy statistics corresponding to the peak stress points. Particularly, the Ud values of the bare coal sample and the coal sample with a thin coating thickness of 0.25 mm remain less than 2.60 kJ/m^3^, suggesting that plastic damage in the pre-peak stage is relatively weak, while the external input energy is mainly accumulated in the form of elastic strain energy. When the coating thickness is further increased to 0.5 mm and 0.75 mm, the corresponding Ud value continues to increase to 4.10 kJ/m^3^ and 5.39 kJ/m^3^, respectively. Conversely, when the coating thickness reaches 1.0 mm or 1.25 mm, the Ud value is reduced to 3.23 kJ/m^3^ and 2.21 kJ/m^3^, respectively, rather than continuing to increase. Meanwhile, the Ue values of coal samples with coating thicknesses of 0.75 mm, 1.0 mm, and 1.25 mm can be determined as 6.77 kJ/m^3^, 8.16 kJ/m^3^, and 7.73 kJ/m^3^, respectively.

The aforementioned results demonstrate that different polyurea coating thicknesses exhibit significant differences in their effects on the pre-peak damage evolution and energy distribution mechanisms of coal samples. For the bare coal samples and the coal samples with a thin coating thickness of 0.25 mm, due to the limited lateral constraint effect, the energy evolution in the pre-peak stage is not significantly altered. Noticeably, when the coating thicknesses reach 0.5 mm and 0.75 mm, the polyurea coatings undergo coordinated deformation and damage with the coal samples, and thus the pre-peak dissipation strain energy is remarkably improved. In contrast, when the coating thickness exceeds 1.00 mm, the initiation and propagation of microcracks within the coal sample are suppressed by the strong constraint effect, and thus the pre-peak damage and energy dissipation are inhibited, while the external work is primarily stored as elastic strain energy within the polyurea-coal composite system.

#### 3.4.3. Energy Evolution Characteristics of Coal Samples with Various Polyurea Coating Thicknesses

**Figure 13:**
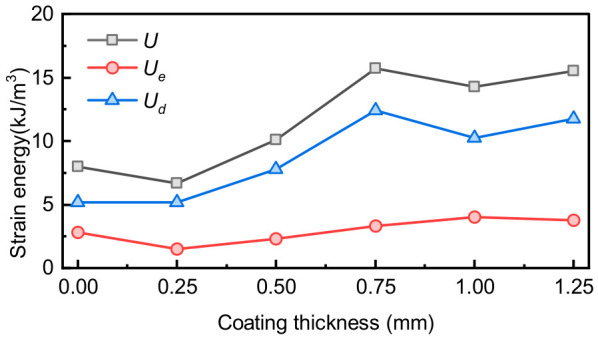
Energy statistics of coal samples with various coating thicknesses in the complete failure stage.

As shown in Figure 13, the statistical analysis of energy data in the complete failure stage of coal sample reveals that unlike the decreasing trend of Ud at the peak stress point, the Ud values of the coal sample with a coating thickness of 1.0 mm and the coal sample with a coating thickness of 1.25 mm in the complete failure stage are increased to 10.27 kJ/m^3^ and 11.75 kJ/m^3^, respectively, which are close to the Ud value of 12.43 kJ/m^3^ of the coal sample with a coating thickness of 0.75 mm. These results demonstrate that although the polyurea coating thickness can significantly alter the stage-wise distribution characteristics of energy dissipation, the total dissipation strain energy of the coal sample tends to stabilize in the complete failure stage when the coating thickness reaches a certain threshold value.

Overall, the temporal characteristics of energy dissipation in the coal sample can be determined by the polyurea coating thickness. When the polyurea coating is relatively thin or of moderate thickness (e.g., 0.75 mm), the coating undergoes coordinated deformation and localized damage in the pre-peak stage, and thus some input energy is dissipated in advance. In contrast, when the coating thickness is relatively large, as the structural integrity and tensile deformation resistance are significantly enhanced, the coal sample is less susceptible to significant damage in the pre-peak stage, and thus more external work is converted into elastic energy stored within the system. When macroscopic fracturing occurs in the post-peak stage, owing to the excellent passive tensile deformation capability, the thick polyurea coating can effectively restrain the fragmentation of coal blocks and expansion, and during this process, a large amount of elastic energy accumulated in the pre-peak stage and the energy continuously input in the post-peak stage are absorbed and dissipated, while the failure mode of instantaneous and intense release of elastic energy is fundamentally altered in the post-peak stage of bare coal sample.

## 4. Discussion

### 4.1. Mechanical Enhancement Mechanism of Coal Mass Constrained by Polyurea Coating

#### 4.1.1. Pseudo-Triaxial Constraint Effect

In Section 3.1, the experimental results demonstrate that with increasing polyurea coating thickness, both the peak stress and peak strain of the coal sample are synchronously enhanced, while the post-peak stress reduction characteristic transitions from a sudden reduction to a gradual decrease. In Section 3.2, the DIC results further reveal that the polyurea coating can effectively inhibit the lateral expansion of the coal sample. Additionally, when the coating thickness is relatively large, a pronounced unloading rebound phenomenon is observed under unloading. These aforementioned phenomena collectively indicate that during the compressive deformation of the coal sample, the passive tensile deformation is induced in the polyurea coating by the lateral expansion of the coal mass, and continuous lateral confinement is imposed on the coal mass, while the original uniaxial compression condition of the coal mass transitions into a pseudo-triaxial stress state. This additional constraint pressure effect is regarded as the mechanical basis for polyurea coating to improve the strength of coal samples, delay instability and enhance ductile failure capabilities.

**Figure 14:**
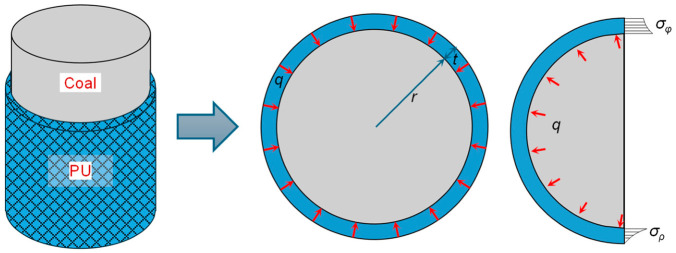
Mechanical enhancement mechanism of coal sample constrained by polyurea coating.

To analyze the constraint effect of polyurea coating on the coal sample, the coal sample with the polyurea coating can be simplified into a mechanical model. As illustrated in Figure 14, the polyurea coating is simplified as a circular ring subjected to uniformly distributed pressure. Specifically, the radius of the coal sample is denoted by r, the thickness of the polyurea coating by *t*, the equivalent constraint pressure at the interface by $q_{a}$, and the polar-coordinate radius by *ρ*. In addition, the circumferential normal stress and radial normal stress are denoted by *σφ* and *σρ*, respectively, while the circumferential shear stress and radial shear stress are denoted by *τφ* and *τρ*, respectively. The corresponding stress boundary conditions are expressed by Equation (4) as follows.

(4) $$\begin{array}{c} {\left(\tau_{\rho \varphi }\right)}_{\rho =r}=0,{\left(\tau_{\rho \varphi }\right)}_{\rho =r+t}=0\\ {\left(\sigma_{\rho }\right)}_{\rho =r}=-q_{a},{\left(\tau_{\rho }\right)}_{\rho =r+t}=0 \end{array}\},$$

where, by substituting the above boundary conditions into the equilibrium equation and solving it using the inverse solution approach, the circumferential stress *σ_φ_* and radial stress *σ_ρ_* of the circular ring can be obtained (Equation (5)).

(5) $$\begin{array}{c} \sigma_{\rho }=-\frac{-1}{{\left(r+t\right)}^{2}∕r^{2}-1}q_{a}\\ \sigma_{\varphi }=\frac{{\left(r+t\right)}^{2}∕\rho^{2}+1}{{\left(r+t\right)}^{2}∕r^{2}-1}q_{a} \end{array}\},$$

Based on the maximum tensile stress *σ_max_* of the polyurea coating, the imposed maximum constraint pressure can be calculated.

Since *σ_φ_* reaches the maximum at the inner boundary of the circular ring, when *ρ* = *a* and *σ_φ_ = σ_max_*, the maximum constraint pressure *q* can be obtained.

(6) $$q=\frac{\sigma_{max}\left(t^{2}+2rt\right)}{{\left(r+t\right)}^{2}+r^{2}},$$

Based on the Mohr–Coulomb criterion, the ultimate compressive strength of the coal sample under compression can be described by Equation (7), as follows.

(7) $$\sigma_{1}=\frac{2c\cos\varphi }{1-\sin\varphi }+\sigma_{3}\frac{1+\sin\varphi }{1-\sin\varphi },$$

where *φ* is the internal friction angle of the coal sample, degrees (°); c is the cohesion of the coal sample, MPa; σ_3_ is the horizontal stress on the coal sample, MPa; and *σ*_1_ is the ultimate compressive strength of the coal sample, MPa.

With the combination of Equations (6) and (7), the strength increment of the coal sample under the imposed constraint can be achieved by Equation (8), as follows.

(8) $$\sigma_{1}=\frac{\sigma_{max}\left(t^{2}+2rt\right)}{{\left(r+t\right)}^{2}+r^{2}}\frac{1+\sin\varphi }{1-\sin\varphi },$$

**Figure 15:**
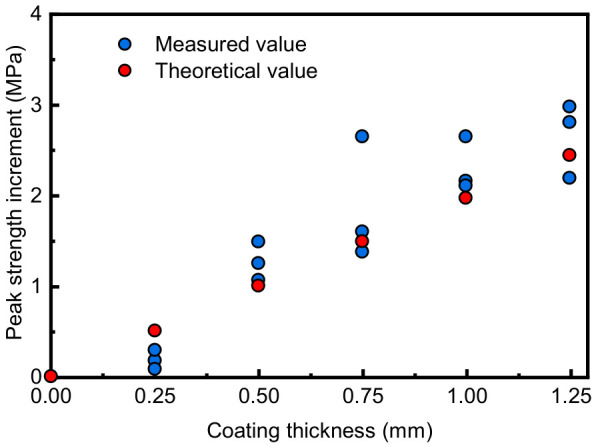
Strength increments of coal samples with various polyurea coating thicknesses compared with the relevant theoretical calculation results.

To quantitatively analyze the constraint effect of polyurea coating on the coal sample, according to the standard coal sample dimensions for uniaxial compression testing and the internal friction angle of commonly used coal, φ = 30° and r = 25 mm. The tensile strength $\sigma_{max}$ of polyurea can be determined as 25.49 MPa. Additionally, the experimentally measured peak stress increments of coal samples with various coating thicknesses were compared with the relevant theoretical calculations, and Figure 15 presents the corresponding results. Particularly, the measured peak strength increment was achieved by subtracting the average peak strength of the bare coal sample from the peak strength of each group (Figure 5a).

As shown in Figure 15, the data demonstrate that with increasing the coating thickness, the theoretical strength increment increases from 0.51 MPa to 2.45 MPa. The experimentally measured peak stress increments of coal samples with various coating thicknesses are highly consistent with the relevant theoretical calculations, but the dispersion is still observed under the same coating thickness. For example, at t = 0.75 mm, the theoretically calculated increment is 1.49 MPa, while the experimentally measured increments for three samples in the same group are 1.38 MPa, 1.60 MPa and 2.65 MPa, respectively. This dispersion is mainly associated with the intrinsic heterogeneity of defects within the coal mass, the localized coating thickness difference and the fluctuation of interfacial bonding state. The consistency between the theoretical prediction and the experimentally measured results in the overall trend validates the rationality of the equivalent lateral constraint model, which also provides a theoretical explanation for the mechanical response of the coal sample constrained by the polyurea coating in Section 3.1.

It should be noted that the equivalent lateral constraint model assumes the coal mass to be an isotropic elastic continuum for analytical tractability. However, coal is inherently anisotropic due to its layered sedimentary fabric and oriented cleat system. The present model does not account for the anisotropic elastic properties or the presence of pre-existing microcracks. These factors introduce uncertainties in the theoretical predictions, as reflected in the discrepancy between the measured and calculated strength increments. Future refinements of the model should incorporate a damage variable representing initial microcrack density to improve prediction accuracy.

#### 4.1.2. Mechanical Mechanism of Tensile-Shear Crack Proportion Transition

In Section 3.3.1, the acoustic emission AF-RA parameter analysis demonstrates that the shear cracks account for a high proportion in the bare coal samples. However, when the polyurea coating thickness is ≥0.50 mm, the proportion of tensile cracks steadily increases to ~90%. Meanwhile, in Section 3.3.2, the b-value and AE energy results further demonstrate that under the constraint effect of thick polyurea coating, the post-peak high-energy fracture events are significantly attenuated, suggesting that the intense shear displacement no longer plays a dominant role within the coal mass. These aforementioned phenomena indicate that the effect of polyurea coating on the coal sample fracture mechanism is not only reflected in the change in the number of cracks but also in the reconstruction of the dominant fracture mode. To reveal the underlying reason, it is essential to analyze this phenomenon from the perspective of the rock shear fracture mechanism.

**Figure 16:**
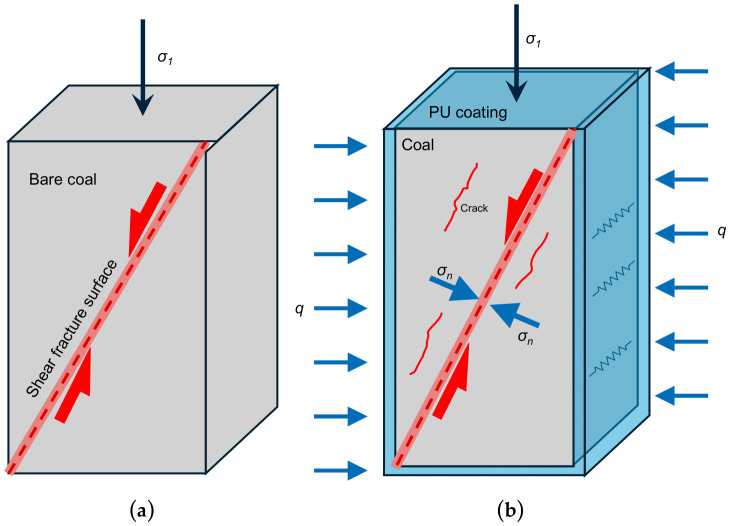
Principle of the variation in shear crack number under the constraint effect of polyurea coating: (**a**) schematic diagram of shear fracture in the bare coal under uniaxial compression; (**b**) constraint mechanism of polyurea coating on the shear fracture of the coal sample.

In addition, the macroscopic shear fracture of the coal mass can often be explained by the Mohr–Coulomb criterion. As illustrated in Figure 16, under uniaxial compression, a shear fracture plane is generated within the coal sample. After the introduction of polyurea coating, the outer material undergoes passive tensile deformation during the lateral expansion process of coal, which can provide equivalent lateral constraint to the coal mass, and thus the normal stress on the potential fracture plane is enhanced. The shear strength τ can be expressed by Equation (9), as follows.

(9) $$\tau =c+\sigma_{n}tan\varphi$$

where *c* is the cohesion, *φ* is the internal friction angle, and *σ*_n_ is the normal stress on the fracture plane.

The equivalent lateral constraint *q* provided by polyurea is regarded as an additional constraint pressure, and then, the shear strength of the potential fracture plane under the constraint effect can be rewritten by Equation (10), as follows.

(10) $$\tau =c+\frac{\sigma_{max}\left(t^{2}+2rt\right)}{{\left(r+t\right)}^{2}+r^{2}}tan\varphi$$

Hence, the corresponding shear strength increment Δ*τ* can be described by Equation (11), as follows.

(11) $$\Delta \tau =\frac{\sigma_{max}\left(t^{2}+2rt\right)}{{\left(r+t\right)}^{2}+r^{2}}tan\varphi =\sigma_{max}tan\varphi \left(1-\frac{{2r}^{2}}{{\left(r+t\right)}^{2}+r^{2}}\right)$$

Based on Equation (11), it can be seen that with the increasing coating thickness, the polyurea coating can provide a more pronounced lateral constraint, and thus the shear strength of the potential fracture plane is significantly improved, while it becomes more difficult for shear slip to occur. This conclusion is highly consistent with the experimental results in Section 3.3.1, and the proportion of shear cracks is progressively reduced from 26.2% in the bare coal sample to ~9–14%, which also provides a mechanistic explanation for the coal sample with the thick coating in Section 3.2.1 maintaining its good integrity after failure. In other words, the introduction of polyurea coating does not simply prevent the occurrence of failure; rather, by increasing the normal stress and shear strength, it inhibits the shear slip and crack propagation, and thus the coal fracture transitions from a highly abrupt shear instability to relatively gradual tensile cracking and progressive propagation.

Furthermore, this mechanism is correlated with the damage evolution characteristics in Section 3.1.1. The bare coal sample exhibits a significant increase in AE ringing counts and abrupt changes in the damage variables near the peak stress, indicating that the rapid internal crack penetration is accompanied by intense energy release. In contrast, the damage variables of the coal sample with the polyurea coating exhibit a gradual accumulation in the post-peak stage, reflecting a transition in the crack propagation mode from instantaneous penetration to continuous evolution. Hence, it is evident that the reconstruction of the tensile–shear crack ratio is the core microscopic mechanism by which the polyurea coating can enhance the strength and alter the failure mode of the coal sample.

It should be emphasized that the passive confinement exerted by the polyurea coating is fundamentally different from the constant or stiff lateral restraint provided by steel jackets or conventional triaxial cells. A rigid vessel typically imposes a fixed-displacement boundary, which can elevate strength but provides negligible energy absorption and may even promote violent energy release after failure of the brittle core. In contrast, the polyurea film undergoes large elastic–plastic tensile deformation synchronously with the coal’s lateral expansion and continues to stretch in the post-peak stage; this not only raises the shear resistance on incipient fracture planes (Equation (11)) but also redistributes the input energy temporally—partially dissipating it through coating deformation before and after the peak, thereby buffering the instantaneous kinetic energy release that is characteristic of bare coal. Such a coupled confinement–energy-buffering function cannot be achieved by stiff liners and is the essential reason why polyurea transforms the catastrophic splitting into a progressive ductile damage mode, as demonstrated in Section 3.1 and Section 3.4. This unique dual role highlights that the present study goes beyond the classic soil/rock–vessel interaction concept and provides a mechanistic basis for designing thickness-adaptive, flexible support systems.

### 4.2. Pre-Peak Elastic Energy Storage and Impact Tendency Evaluation

In Section 3.4, the energy analysis demonstrates that the polyurea coating not only alters the strength and failure mode of the coal sample but also changes the distribution ratio of pre-peak input energy accumulation and post-peak energy release. Specifically, under a coating thickness of 0.50–0.75 mm, the pre-peak dissipation strain energy is remarkably increased, indicating that the polyurea coating can dissipate some input energy in advance through the coordinated deformation and localized damage with the coal sample. In contrast, when the coating thickness is ≥1.00, the pre-peak dissipation strain energy is reduced, but the total dissipation strain energy in the complete failure stage is increased again, indicating that the relatively thick polyurea coatings are mainly dependent on the large post-peak deformation to absorb and buffer elastic energy. This demonstrates that the weakening of impact tendency by the polyurea coating is essentially derived from its regulatory effect on the process of pre-peak elastic energy accumulation and post-peak energy release.

The elastic energy index ($W_{ET}$) is commonly adopted to characterize the impact tendency. This parameter can be calculated on the basis of the stress–strain curve as the ratio of the elastic strain energy before instability${\;U}_{e}$ to the plastic deformation energy $U_{d}$ [52,53]. Based on the magnitude of $W_{ET}$, the impact tendency of the coal sample can be classified into three levels, including non-impact, weak-impact, and strong-impact. Specifically, when $W_{ET\;}$ < 2, it represents the non-impact tendency. When $2\leq W_{ET}$ < 5, it represents the weak-impact tendency. When $W_{ET}\geq 5$, it represents the strong-impact tendency. The corresponding calculation can be conducted by Equation (12), as follows.

(12) $$W_{ET}=\frac{U_{e}}{U_{d}}$$

**Figure 17:**
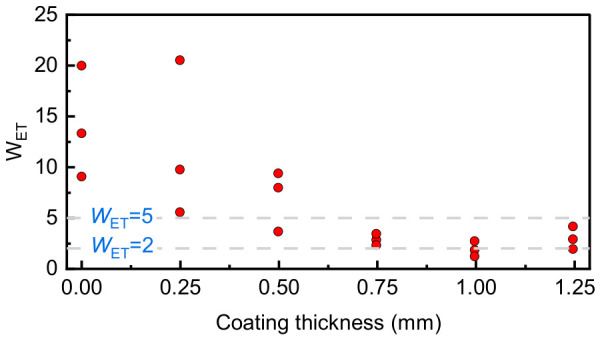
Elastic energy index ($W_{ET}$) of coal samples with various polyurea coating thicknesses.

As shown in Figure 17, the $W_{ET}$ values of the coal samples exhibit significant changes under different polyurea thicknesses, while an overall decreasing trend can be observed with increasing coating thickness. For the bare coal sample without polyurea coating (0 mm), the WET values of the three sample groups are higher than 5, which can be determined as 13.278, 9.005, and 19.960, indicating a strong impact tendency. This is highly consistent with the phenomena in Section 3.1.1, including the rapid reduction in the post-peak stress in the bare coal sample, the abrupt change in the damage variables, and the instantaneous surge in AE ringing counts, demonstrating that a large amount of releasable elastic strain energy accumulates and releases upon instability in the bare coal sample.

When the polyurea coating thickness reaches 0.25 mm, the WET value remains within the range of 5.464–20.500, and all three sample groups exhibit a strong impact tendency, suggesting that the thin polyurea coating has a limited effect on reducing the impact tendency. This is also consistent with the results in Section 3.2.1, and the coal sample with a coating thickness of 0.25 mm still exhibits obvious surface cracks and localized cracking, which is also consistent with the results in Section 3.4.2, while the pre-peak energy dissipation improvement is relatively limited.

As the coating thickness is increased to 0.50 mm, the WET value is reduced to 3.542–9.316, and the impact tendency begins to differentiate, demonstrating that samples with both strong and weak impact tendencies can be obtained. This is consistent with the results in Section 3.3.1, and the proportion of tensile cracks is remarkably increased under this thickness condition. In Section 3.4.2, the pre-peak dissipation energy is significantly improved, suggesting that polyurea coating begins to exert a regulatory effect on the energy accumulation and dissipation mechanism, but it is still affected by the sample dispersion, interfacial bonding state, and the distribution of intrinsic defects.

In addition, when the polyurea coating thickness is further increased to 0.75 mm, the WET values of the three sample groups are within the range of 2–5, which can be determined as 2.727, 3.319 and 2.191, respectively, revealing a weak impact tendency. This result is completely consistent with the finding in Section 3.4.2, and the coal sample with the polyurea coating thickness of 0.75 mm exhibits the highest pre-peak energy dissipation. It demonstrates that this polyurea coating thickness is most effective in pre-peak synergistic energy dissipation, which can significantly increase the proportion of dissipated strain energy and reduce the elastic energy storage. Therefore, the polyurea coating thickness of 0.75 mm can be regarded as the critical thickness for achieving a stable transition from a strong-impact to a weak-impact tendency in the coal samples.

When the polyurea coating thicknesses reach 1.00 mm and 1.25 mm, their average WET values are less than 2, indicating the lowest impact hazard. With the combination of the unloading rebound phenomenon in Section 3.2.3 and the significantly increased energy dissipation in the complete failure stage in Section 3.4.3, it can be seen that the advantage of polyurea under these conditions is no longer reflected in pre-peak energy dissipation but rather in the post-peak absorption of fracture-released energy through the high ductility and elastic deformation capacity, which is conducive to buffering the block ejection and crack propagation.

### 4.3. Influence of Natural Heterogeneity of Coal Mass on Coating Effectiveness

While the equivalent lateral constraint model in Section 4.1 effectively reveals the macroscopic strengthening mechanism of the polyurea coating, coal is inherently a highly heterogeneous geomaterial containing natural matrix pore structures, multi-scale cleats, and microcracks. This intrinsic heterogeneity significantly influences both the failure evolution and the efficiency of the flexible coating.

First, the distribution of natural macro- and micro-defects dictates the localized mechanical response of the coal mass. During initial compression, primary internal cracks act as stress-concentrators. Unlike ideal homogeneous materials, coal is prone to being locally intensified by preferential sliding along natural weak cracks or planes. Consequently, the uniform external polyurea coating is subjected to non-uniform local tension. As shown in the horizontal displacement contour maps (Figure 7), the coating efficiency depends on its capability to bridge these locally localized high-strain zones. Thicker coatings (≥0.75 mm) are required to thoroughly neutralize the non-uniform transverse expansion induced by structural micro-uniformities, avoiding premature localized tearing.

Second, the structural heterogeneity objectively leads to variability in macroscopic mechanical properties. As presented in Figure 15, the experimentally measured peak stress increments exhibit noticeable intra-group dispersion compared to the theoretical model (e.g., at t = 0.75 mm, the increments vary from 1.38 MPa to 2.65 MPa). Similarly, the elastic energy index (W_ET_) in Figure 17 exhibits wide fluctuations under the identical coating condition. This variability demonstrates that the overall failure picture of the material is a superposition of the coating’s constraint and the random original defect distribution. If a critical natural macroscopic fracture happens to traverse the entire sample randomly, the flexible coating can predominantly delay complete failure but may struggle to fully reverse the instantaneous internal dynamic release.

Moreover, the heterogeneity of the coal surface critically affects the formation and continuous operational effectiveness of the polyurea layer. The natural coal surface morphology, characterized by uneven micro-roughness and variable porosity, directly influences the interfacial wetting and anchoring characteristics. Despite pre-cleaning with ethanol, varying levels of localized surface pulverization and micro-dust inevitably remain. These structural features can cause localized fluctuations in adhesion strength (Table 1). This phenomenon fully explains the macroscopic observations in Section 3.2.1, where minor cracking and spalling initially occurred at the sharp edges of the coal specimens. At these locations, the geometric singularities coupled with localized weaker interfacial adhesion compromised the uniformity of the protective layer.

### 4.4. Limitations and Future Work

In this study, the regulatory effects of polyurea coatings on the macroscopic mechanical behavior, microscopic fracture mechanism, and energy dissipation characteristics of coal samples were systematically elucidated. Nevertheless, with the contribution of the above discussion, it can be seen that the present research still has certain limitations in terms of sample configuration, loading path and engineering applicability, which can be further improved in future work.

First, in this study, an ideal uniform constraint model based on a cylindrical sample was utilized in the theoretical analysis, whereas the actual experimental samples were selected as standard cuboid coal samples. In Section 3.2.1, it is evident that the edges and ends of cuboid coal samples are susceptible to cracking and spalling, demonstrating that geometric boundary effects significantly impact the uniformity of polyurea constraint. Furthermore, the present theoretical model cannot adequately account for the deviations caused by the stress concentration at corners and localized uneven coating thickness. Additionally, the intrinsic natural cleats and microcracks within the coal rocks exhibit significant randomness, which can be regarded as one of the primary reasons for the intragroup dispersion of strength increment in Section 3.1.2 and the elastic energy index WET in Section 4.2. Hence, future research should expand the sample size and adopt the standard cylindrical samples for comparative studies to further improve the consistency between the theoretical model and experimental results. Moreover, the combined effects of chemical corrosion, long-term moisture ingress, and elevated temperature may degrade the interfacial adhesion between polyurea and coal, as well as the intrinsic mechanical properties of the coating itself. Therefore, systematic durability tests under in-situ environmental coupling are necessary to translate the short-term laboratory-observed benefits into long-term underground support designs.

Second, in this study, the primary emphasis was placed on the static uniaxial compression conditions. However, rock bursts and impact ground pressure disasters in deep mines generally occur under complex stress environments with the synergistic effects of high static loads and dynamic disturbances. The strengthening and energy regulation mechanisms of polyurea constraint revealed in Section 3 are primarily applicable to the mechanistic explanations under static loading conditions, but they are still insufficient to fully reflect the actual response of deep coal and rock under high constraint pressure, cyclic disturbances and transient impact loads. To further validate the lateral constraint–crack mechanism transition–energy path reconstruction framework proposed in this work, cyclic loading–unloading tests, true triaxial compression tests, and Split Hopkinson Pressure Bar (SHPB) experiments should be conducted in future research, and thus, the dynamic toughening and anti-impact performance of polyurea under complex stress paths can be elucidated.

The idealized testing environment under laboratory conditions cannot encompass all the various service factors encountered in deep engineering sites. In actual mining environments, there are generally adverse conditions, such as high temperature, high humidity, groundwater-induced erosion, and surface pulverization of coal walls, which may weaken the interfacial bonding performance between the polyurea coating and the coal mass, and the continuous stability of the constraint effect can be further affected. Meanwhile, the unloading rebound phenomenon of thick polyurea coating in Section 3.2.3 also demonstrates that the polyurea coating may exhibit a significant time-dependent effect and creep response under long-term loading conditions. Accordingly, the interfacial debonding and failure mechanisms of the polyurea coating–coal rock interface, the durability evolution under environmental coupling effects, and the constraint attenuation under long-term service conditions will become crucial research directions for advancing the field-scale engineering implementation of this technology.

The accuracy and reproducibility of experimental results are inherently constrained by the inability to completely control or accurately quantify the initial internal structural state of raw coal samples. The current methodology relies primarily on macroscopic parameters (stress–strain) and external surface characterizations (DIC and AE). Because pre-test non-destructive 3D quantitative mapping (such as X-ray micro-computed tomography) was not performed, the precise correlation between the spatial distribution of internal initial cleats and the localized failure efficiency of the polyurea layer remains an evaluation “black box”. Future work must establish a collaborative evaluation framework incorporating X-ray 3D-CT to precisely isolate the variables of original defect configurations from the coating reinforcement effects, thus narrowing the error boundaries.

Furthermore, the present study was conducted on intact and dry coal specimens. However, in situ coal masses ubiquitously contain pre-existing open cracks, weak interlayers, and varying degrees of water saturation, which may significantly modulate the effectiveness of the polyurea coating. For coal masses with extensive pre-existing open fractures, the continuity and uniformity of the passive lateral confinement could be compromised, as the coating cannot bridge or reinforce these voids; deformation concentrations around crack tips may cause premature local coating rupture or interfacial debonding, thereby diminishing its capacity to suppress lateral expansion and alter the fracture mode. In the presence of weak interlayers, the failure plane may be dictated by the weakest geological interface rather than by the stress state within the intact coal. Even with increased normal stress from the coating, shear slip along the interlayer could still dominate, potentially offsetting the coating-induced transition from shear- to tension-dominated micro-fracturing observed in our experiments. Water saturation introduces additional complexity: while polyurea itself is highly resistant to water and hydrolysis, its adhesion strength to the coal substrate can be reduced by pore-water pressure at the interface. Moreover, the inherent strength and brittleness of water-saturated coal are altered, which would undoubtedly shift the energy partitioning and impact proneness profiles we identified. A systematic investigation into how these ubiquitous geological and environmental factors interact with the polyurea confinement mechanism constitutes a critical and necessary step for advancing this technology from laboratory validation to reliable field-scale engineering design.

## 5. Conclusions

In this study, with a combination of the uniaxial compression test, digital image correlation (DIC) and acoustic emission (AE) techniques, the regulatory effects of polyurea coatings on the mechanical strength, microscopic fracture mechanism, and energy evolution of coal samples were systematically elucidated. The main conclusions can be drawn as follows.

(1) The introduction of polyurea coating can significantly increase the macroscopic mechanical strength of the coal mass and improve its fracture characteristics through the lateral passive constraint effect. The uniaxial compression results and the DIC full-field displacement cloud maps demonstrate that with increasing polyurea coating thickness, the peak strength of the coal sample is significantly enhanced, while the failure mode transitions from brittle failure to ductile failure. When the coating thickness is relatively large, the polyurea coating can drive the fragmented coal blocks to elastically rebound after unloading. The underlying mechanical mechanism of this phenomenon can be ascribed to the fact that when the coal sample undergoes lateral expansion during the loading process, continuous lateral constraint pressure is imposed on the coal sample by the polyurea coating through its own passive tensile deformation, and thus, the lateral deformation of the coal sample and the propagation of macroscopic cracks are effectively suppressed.

(2) Under the lateral constraint effect of polyurea coating, the microscopic fracture mode of the coal mass transitions from the internal shear fracture to tensile fracture. The quantitative analysis of acoustic emission AF-RA parameters demonstrates that when the coating thickness reaches 0.50 mm or above, the proportion of internal tensile cracks in the coal sample increases from 73.8% of the bare coal sample and stabilizes at ~90%. The fundamental reason for this microscopic mechanism transition is derived from the lateral constraint effect of polyurea coating, significantly increasing the normal stress on the potential fracture plane within the coal mass, and thus the shear strength of the coal mass is remarkably enhanced.

(3) The energy dissipation behavior of coal and rock can be further regulated by the polyurea coating through altering the microscopic fracture path. The data analysis shows that during the loading process, the polyurea coating undergoes coordinated deformation with the coal sample, and a portion of the input energy is effectively absorbed. Notably, when the coating thickness reaches 1.00 mm or above, the energy absorption performance is significantly improved. Particularly, in the post-peak fracture stage of the coal sample, owing to the excellent tensile deformation capacity, the polyurea coating can absorb and buffer a large amount of elastic energy released by the fracture of the coal sample. This phenomenon suggests that the highly elastic and plastic polyurea coating plays a buffering role during the failure process of coal and rock, and the post-peak instantaneous and intense release mode of elastic energy in the bare coal sample is altered.

(4) The impact tendency of the coal sample is effectively weakened by the introduction of polyurea coating through the alteration of the energy release behavior of coal and rock during failure. The analysis of the elastic energy index (WET) validates that when the coating thickness reaches 0.75 mm or above, the impact tendency of the coal sample can be steadily reduced from the strong-impact tendency of the bare coal sample to a range of weak-impact or even non-impact tendency. This phenomenon can be ascribed to the fact that the polyurea coating can effectively increase the proportion of dissipated strain energy within the coal sample, while the accumulation of elastic energy is dramatically reduced within the coal sample, and thus the instantaneous energy release during the coal and rock instability process is fundamentally inhibited.

Overall, this study demonstrates that a flexible polyurea coating is an effective surface-confinement strategy for regulating fracture and energy release in brittle coal.

## Figures and Tables

**Table 1:** Comparative analysis of key parameters for TSLs applied in underground coal/rock mass.

Type of Coating	Tensile Strength (MPa)	Elongation at Fracture (%)	Adhesion Strength (MPa)	Curing Time	Sensitivity to External Humidity
Cementitious TSL [27,28,29]	3.7~8.1	—	0.7–1.8	7~28 day	High
Epoxy Resin [30,31,32]	55~81	~5.3	>1.5	—	High
Polyurethane (PU) [33,34,35,36]	13~19.5	495~650	<4	—	Low
Polyurea (PUA) [37,38,39]	7.5~28	>20	>4	60~180 s	Low

**Table 2:** Fundamental parameters of polyurea coating material.

Parameter	Unit	Value
Drying time	h	4.0
Impact resistance	Kg·cm	100.0
Adhesion strength	MPa	9.0
Tensile strength	MPa	25.5
Elongation at fracture	%	247.0
Tear strength	kN/m	84.3

**Table 3:** Grouping and designations of coal samples.

Group	Series Label	Specimen Description	Coating Thickness (mm)	Specimen Designation
Control group	C	Bare coal specimens	0.00	C1, C2, C3
Experimental group	Pu1	Coal samples under the polyurea coating constraint	0.25	Pu1-1, Pu1-2, Pu1-3
	Pu2		0.50	Pu2-1, Pu2-2, Pu2-3
	Pu3		0.75	Pu3-1, Pu3-2, Pu3-3
	Pu4		1.00	Pu4-1, Pu4-2, Pu4-3
	Pu5		1.25	Pu5-1, Pu5-2, Pu5-3

## Data Availability

The data presented in this study are available upon request from the corresponding author.

## Abbreviations

The following abbreviations are used in this manuscript:

AE	Acoustic Emission
DIC	Digital Image Correlation
PDR	Drilling Pressure Relief
ISRM	International Society for Rock Mechanics
AF	Average Frequency
RA	Rise Time/Amplitude
G-R	Gutenberg–Richter
WET	Elastic Energy Index
SHPB	Split Hopkinson Pressure Bar
